# Sarcoma of unknown primary: myth or reality?

**DOI:** 10.1186/s43046-022-00128-1

**Published:** 2022-06-27

**Authors:** Elie Rassy, Rosy Abou-Jaoude, Stergios Boussios, Tarek Assi, Joseph Kattan, Hussein Khaled, Nicholas Pavlidis

**Affiliations:** 1 Department of Medical Oncology, Institut Gustave Roussy, Villejuif, France; 2 Department of Pathology, Saint Joseph University, Beirut, Lebanon; 3 King’s College London, School of Medicine, Guy’s campus, London, SE1 9RT UK; 4 Medway NHS Foundation Trust, Windmill Road, Gillingham, Kent, ME7 5NY UK; 5 AELIA Organization, 9th Km Thessaloniki-Thermi, 57001 Thessaloniki, Greece; 6 Department of Medical Oncology, Saint Joseph University, Beirut, Lebanon; 7 Department of Medical Oncology, National Cancer Institute, Cairo University, Cairo, Egypt; 8 University of Ioannina, Ioannina, Greece

**Keywords:** Cancer of unknown primary, Sarcoma, Soft-tissue sarcoma of unknown primary

## Abstract

**Background:**

Sarcoma of unknown primary (SUP) designates an enigmatic entity with histologic confirmation of a metastatic tumor without an identifiable primary after a thorough diagnostic workup. The term “unknown primary” is heavily debatable given that sarcomas can arise from any tissue that harbors its histological structure. In this review, we discuss the validity of SUP as a distinct entity.

**Main body of the abstract:**

Medline/PubMed and Google Scholar were searched from 1990 until April 2020 for publications in the English language reporting on SUP. We excluded articles reporting on cases with sarcomas from known organ sites such as lung or uterine sarcomas as well as synovial sarcomas. The Kaplan–Meier method was used to compute the median overall survival. A total of 26 patients with SUP were identified. The median age at diagnosis was 17.5 years with a similar prevalence among men and women. The tumors most commonly reported were alveolar rhabdomyosarcoma and rhabdomyosarcoma not otherwise specified. Almost two-thirds of the patients were reported to have more than one metastatic site. Among the 13 patients with survival data, the median overall survival was 10.0 months. Two patients underwent autopsy and had their primary culprit identified in the chest wall and paravertebral.

**Conclusions:**

This review showed that SUP shares with sarcomas of known primary similar clinical features including an aggressive clinical course, generally poor response to chemotherapy, and dismal patient outcomes. Thus, SUP does not appear to display a different natural history and biological properties that would allude to a distinct entity.

**Supplementary Information:**

The online version contains supplementary material available at 10.1186/s43046-022-00128-1.

## Background

Sarcomas enclose a group of heterogeneous malignancies that constitute approximately 1% of human malignancies [1]. These tumors are traditionally categorized according to their clinical presentation into low-grade tumors with minimal metastatic potential and highly aggressive cancers with a tendency for systemic metastasis [2]. The molecular advances over the last two decades identified two groups of sarcomas. The first group is characterized by a tumor-specific translocation that is central to sarcomagenesis and the second presents genetic instability that manifests in a complex karyotype [3]. Both subsets can arise for the mesenchymal tissue in almost every organ and can lead to soft tissue tumors at any site without being attributed to any organ [4, 5]. Although sarcomas arise in tissues of mesenchymal lineages including bone, muscle and cartilage, several publications have reported on the occurrence of sarcomas in the absence of an identifiable primary (supplementary material). Historically, sarcomas of unknown primaries (SUP) accounted for approximately 3.2% of cancers of unknown primary although the term “unknown primary” is heavily debatable among sarcoma experts given that sarcomas of different types can arise from any organ or tissue that harbors its histological structure [6]. For instance, the term synovial sarcoma seems to be carried over from older literature that diagnosed synovial differentiation based on the propensity for this malignancy to originate in periarticular regions and the presence (especially in biphasic cases) of some reminiscent histology [7]. The traditionally reported synovial sarcomas of unknown primary are in fact synovial sarcomas of unusual sites. Therefore, we performed a systematic review and individual-based meta-analysis to assess and discuss whether SUP may be considered as a valid distinct entity.

## Main text

### Methods

Medline/PubMed and Google Scholar were searched from 1990 until April 2020 for publications in the English language reporting on SUP. The search was carried out using mainly (“Sarcoma”[Mesh]) AND “Neoplasms, Unknown Primary”[Mesh]) in Medline or the following keywords such as “Sarcoma”, “Soft-tissue sarcoma”, “Cancer of Unknown Primary” in Google Scholar. Two medical oncologists (ER and NP) reviewed the publication titles and abstracts for relevance and then assessed the references of these papers to ensure the exhaustiveness of the selection process. A pathologist (RAJ) also reviewed the selected cases to confirm eligibility. We excluded articles reporting on cases with synovial sarcomas occurring in unusual sites according to the world health classification of soft tissue tumors published in 2020 [8]. These sites included external and internal reproductive organs, kidney, adrenal gland, retroperitoneum, stomach, small bowel, lung, heart, mediastinum, bone, central nervous system, and peripheral nerve. The individual-patient data reported were extracted from the selected papers and entered into an excel sheet for analyses (supplementary material). Descriptive statistics were used to report on the patient and tumor characteristics, including age, gender, metastatic sites, and diagnostic workup. The Kaplan–Meier method estimated the overall survival defined from the date of diagnosis to the date of death from all causes; the patients who were alive at the time of publication were censored. We did not perform univariable or multivariable analysis given the small number of patients with survival outcomes (*n* = 13). All statistical analyses were performed using IBM© SPSS© Statistics version 26.

## Results

**Fig. 1:**
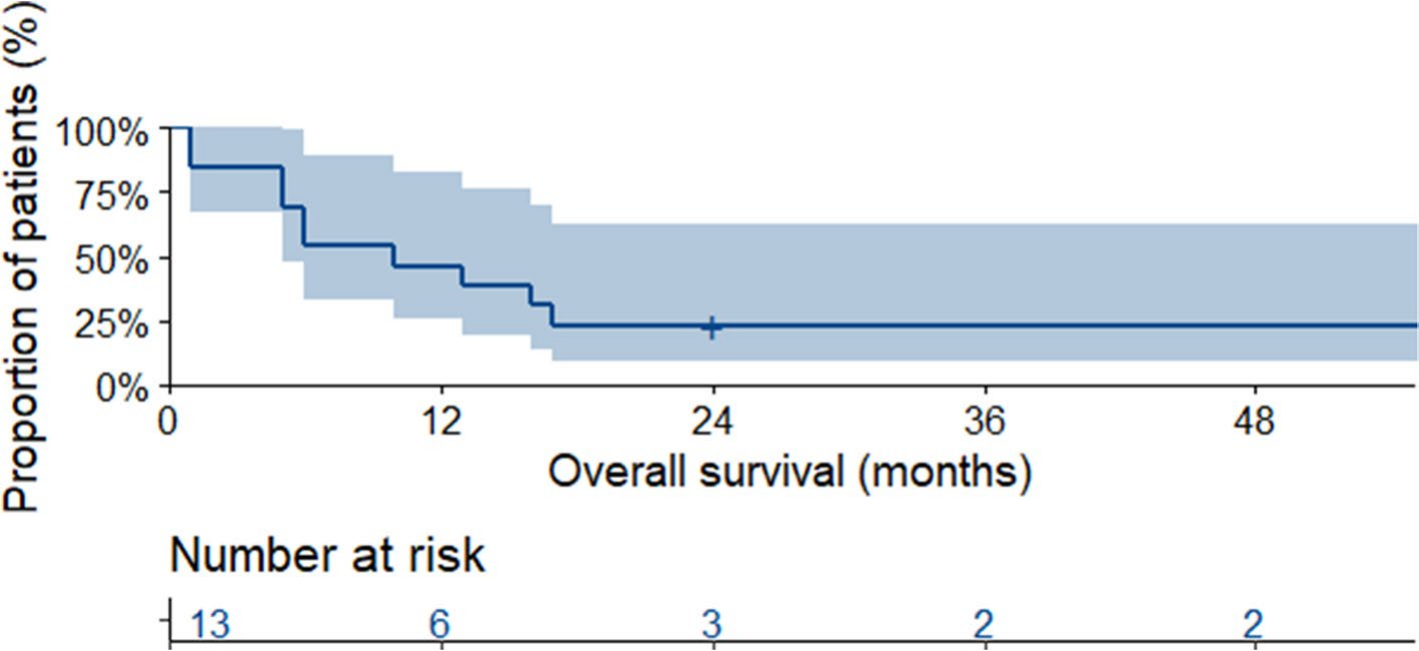
The survival analysis of the 13 patients with available survival data

Overall, 26 patients fulfilled the eligibility criteria for this study and were eligible for analysis. The median age at diagnosis was of 17.5 years (range 3–83 years) with a similar prevalence among men and women. The commonly reported pathologies were alveolar rhabdomyosarcoma (*n* = 7; 26.9%), rhabdomyosarcoma not otherwise specified (*n* = 7; 26.9%), and sarcoma not otherwise specified (*n* = 3; 11.5%). Almost two-thirds of the patients had 2 or more metastatic sites including mainly the brain, bone (including bone marrow), lung, and lymph nodes in 16.7%, 68.8%, 52.9%, and 29.4%, respectively (Table 1). All patients underwent standard morphology and immunohistochemistry studies whereas only four cases had molecular pathology investigations. Among the 11 cases reporting on the diagnostic workup, MRI was used in 18.2%, bone scan in 45.5%, and PET-CT scan in 27.3%. Systemic chemotherapy was administered in 88.2% of patients with only two cases receiving more than one line of chemotherapy. The chemotherapy regimens used were mainly sarcoma-type chemotherapies such as vincristine, topotecan plus cyclophosphamide, doxorubicin plus cyclophosphamide, or vincristine, doxorubicin plus cyclophosphamide. The survival analysis of the 13 patients with available survival data showed a median overall survival of 10.0 months (95% CI 2.9–17.0 months) and 1-year overall survival of 50% (Fig. 1). Two patients underwent autopsy and had their primary culprit identified in the chest wall and paravertebral space.

**Table 1:** Summary of the patients characteristics, diagnostic workup, and treatment modalities

Patients characteristics (*n*)		Number of patients (%)
Gender (*n* = 20)	Male	10 (50.0%)
	Female	10 (50.0%)
Age (*n* = 20)	Median	17.5 years
	Range	3–83 years
Pathology (*n* = 26)	Alveolar rhabdomyosarcoma	7 (26.9%)
	Rhabdomyosarcoma not otherwise specified	7 (26.9%)
	Sarcoma not otherwise specified	3 (11.5%)
	Endometrial stromal sarcoma	2 (7.7%)
	Others^a^	7 (26.9%)
More than one metastatic sites (*n* = 17)		11 (64.7%)
Diagnostic workup	MRI (*n* = 11)	2 (18.2%)
	Bone scan (*n* = 11)	5 (45.5%)
	PET/CT scan (*n* = 11)	3 (27.3%)
Treatment modalities reported	Surgery (*n* = 4)	2 (50.0%)
	Radiotherapy (*n* = 4)	1 (25.0%)
	Chemotherapy (*n* = 17)	15 (88.2%)

^a^Others include dermatofibrosarcoma protuberans (*n* = 1), desmoplastic small round cell tumor (*n* = 1), epithelioid angiosarcoma (*n* = 1), epithelioid sarcoma (*n* = 1), Ewing’s sarcoma (*n* = 1), rhabdomysarcoma undifferentiated (*n* = 1), spindle cell sarcoma (*n*=1)

*n* denotes the number of patients with available information

## Discussion

The historical definition of cancers of unknown primary was based on clinical findings of a multiple tumors site without an identifiable primary and did not require histologic confirmation of the malignancy. This approach was quickly abandoned to the current strategies which mandate pathologic confirmation of the malignancy to guide treatment decisions [9, 10]. The occurrence of SUP is not theoretically valid because sarcomas can arise from any primitive mesenchymal cells and thus in any organ. However, given the complexity and heterogeneity of sarcomas misdiagnosis and incomplete diagnostic workups are not uncommon. From our experience with patients diagnosed with cancers of unknown primary and sarcomas consulting for second opinion at our centers, the diagnostic workups differ widely among centers and patients. For instance, the European Society of Medical Oncology (ESMO) guidelines for sarcoma recommend a centralized pathology review because major discordances occur in 8 to 11% and minor discordances in 16 to 35% [11, 12]. The pathologies of the patients diagnosed with SUP were highly variable reflecting a histologic heterogeneity that complicates diagnostic confirmations (Table 1). In a case series of 96 nonrandomly selected cases with CUP, the accuracy of cytology and transmission electron microscopy in diagnosing tumor category using biopsy results as the gold standard was 78% and 91%, respectively [13]. Among the three cases with sarcoma (2 patients with leiomyosarcoma and 1 with malignant fibrous histiocytoma), the accuracy for diagnosing tumor type was 67% by cytology and 100% by transmission electron microscopy [13]. Moreover, the ESMO guidelines favor molecular diagnostic studies among cases with unusual clinical pathological presentations, thus their indication among patients misdiagnosed with SUP [14]. Only a minority of the patients diagnosed with SUP had molecular pathology investigations although molecular studies were diagnostic in many patients with synovial sarcoma that would be initially considered SUP [15, 16].

The mandatory diagnostic standard for cancers of unknown primary laid down by the European Society of Medical Oncology guidelines includes a CT scan of the chest, abdomen, and pelvis that seemed to be left out in many patients (Table 1). The delicate aspect of this topic is to judge whether a tumor site should be considered the primary culprit or metastatic dissemination to guide treatment decisions. The possible theoretical explanation model for the SUP phenomenon is the smallness of the primary tumor that evades detection or inadequate workup. For example, two patients included in this study underwent autopsy and had their primary culprit identified in the chest wall and paravertebral space. As almost two-thirds of the patients had more than one metastatic site, systemic chemotherapy was the main treatment strategy. Nevertheless, chemotherapy was commonly limited to one line of treatment and the overall survival was dismal. The METASARC observation study reported similar outcomes among 2225 patients with metastatic soft-tissue sarcomas treated in the real-life setting [17]. The median number of systemic treatments was 3 (range, 1–6) with 27% of the patients not receiving any systemic treatment [17]. Almost half the patients underwent locoregional treatment of the metastasis. The median overall survival ranged between 5.4 and 8.5 months and correlated to gender, leiomyosarcoma histologies, locoregional treatment of metastases, and treated with polychemotherapy [17]. Unfortunately, the sample size of patients with SUP reported in the literature was small which limited correlation studies.

## Conclusions

This review showed that SUP shares with sarcomas of known primary similar clinical features, including aggressive clinical course, generally poor response to chemotherapy, and dismal patient outcomes. Thus, SUP does not appear to display a different natural history and biological properties that would allude to a distinct entity. However, this study includes several limitations that are inherent to the study design. The major difficulty encountered in reviewing the literature involved the modifications in the diagnostic criteria of sarcomas. Morphology and immunohistochemistry analysis have been occasionally a topic of disagreement among pathologists which required ancillary molecular diagnostics. Unfortunately, the published literature, mainly the older publications, lack molecular pathology investigations whereas the general approach consists of classifying sarcomas according to their genomic characteristics as either sarcoma with a complex genomic profile or sarcoma with a single underlying (or driver) genetic or molecular-biological abnormality (including translocation, gain-of-function mutation, amplification, or tumor suppressor gene loss) [18–21]. We believe that the small sample and heterogeneity of the study limited our analysis. Large well-validated population-based registries, such as the Surveillance, Epidemiology, and End Results (SEER) program, and more importantly large sarcoma network-based registries should be investigated to confirm whether SUP remains a myth.

## Supplementary Information

**Additional file 1: Supplementary Table 1.** Studies included in this analysis.

## Data Availability

The datasets used and/or analyzed during the current study are available in the supplementary material.

## Abbreviations

SUP: Sarcoma of unknown primary
CT: Computed tomography
