# Supplementary material for: Sarcoma of unknown primary: myth or reality?
Source: J Egypt Natl Canc Inst. 2022 Jun 27;34:27. doi: 10.1186/s43046-022-00128-1 (PMC13314235; doi:10.1186/s43046-022-00128-1)
Supplement: Supplementary file 1 — Additional file 1: Supplementary Table 1. Studies included in this analysis. [file 43046_2022_128_MOESM1_ESM.docx]

| Supplementary Table 1: Studies included in this analysis | | |
| --- | --- | --- |
| Author | N | Pathology details |
| Patiroglu et al. (2014) (1) | 1 | Alveolar rhabdomyosarcoma |
| Jani et al. (2008) (2) | 1 | Alveolar rhabdomyosarcoma |
| Lund et al. (2005) (3) | 1 | Epithelioid angiosarcoma |
| Mentzel et al. (1998) (4) | 1 | Dermatofibrosarcoma protuberans (fibrosarcomatous variant) |
| Allesandrini et al. (2017) (5) | 1 | Endometrial stromal sarcoma |
| Sachpekidis et al. (2016) (6) | 1 | Rhabdomysarcoma undifferentiated |
| Pimiento et al. (2007) (7) | 1 | Sarcoma NOS |
|  | 1 | Sarcoma NOS |
|  | 1 | Sarcoma NOS |
| Motzer et al. (1995) (8) | 1 | Endometrial stromal sarcoma |
| Rong et al. (2009) (9) | 1 | Alveolar rhabdomyosarcoma |
| Nakada et al. (1990) (10) | 1 | Rhabdomysarcoma NOS |
|  | 1 | Rhabdomysarcoma NOS |
| Kuttesch et al. (1995) (11) | 1 | Alveolar rhabdomyosarcoma |
|  | 1 | Alveolar rhabdomyosarcoma |
|  | 1 | Rhabdomysarcoma NOS |
|  | 1 | Alveolar rhabdomyosarcoma |
|  | 1 | Rhabdomysarcoma NOS |
|  | 1 | Alveolar rhabdomyosarcoma |
|  | 1 | Rhabdomysarcoma NOS |
|  | 1 | Ewing sarcoma |
| Backer et al. (1998) (12) | 1 | Desmoplastic small round cell tumor |
| Morandi et al. (1996) (13) | 1 | Rhabdomysarcoma NOS |
| Fitzmaurice et al. (1991) (14) | 1 | Rhabdomysarcoma NOS |
| Yim et al. (1999) (15) | 1 | Spindle cell sarcoma |
| Kuhel et al. (1997) (16) | 1 | Epithelioid sarcoma |
| N: number of patients; NOS: not otherwise specified | | |

1. Patiroglu T, Isik B, Unal E, Canoz O, Deniz K, Kosemehmetoglu K, et al. Cranial metastatic alveolar rhabdomyosarcoma mimicking hematological malignancy in an adolescent boy. Childs Nerv Syst. oct 2014;30(10):1737‑41.

2. Jani P, Ye CC. Massive bone marrow involvement by clear cell variant of rhabdomyosarcoma with an unusual histological pattern and an unknown primary site. J Clin Pathol. févr 2008;61(2):238‑9.

3. Lund L, Amre R. Epithelioid angiosarcoma involving the lungs. Arch Pathol Lab Med. janv 2005;129(1):e7-10.

4. Mentzel T, Beham A, Katenkamp D, Dei Tos AP, Fletcher CD. Fibrosarcomatous (« high-grade ») dermatofibrosarcoma protuberans: clinicopathologic and immunohistochemical study of a series of 41 cases with emphasis on prognostic significance. Am J Surg Pathol. mai 1998;22(5):576‑87.

5. Alessandrini L, Sopracordevole F, Bertola G, Scalone S, Urbani M, Miolo G, et al. Primary extragenital endometrial stromal sarcoma of the lung: first reported case and review of literature. Diagn Pathol. 2 mai 2017;12(1):36.

6. Sachpekidis C, Langer R, Kollàr A, Wartenberg J. Detection of a primary tumor in the area of the renal artery with 18F-FDG PET/CT in a patient with metastatic undifferentiated sarcoma and a history of mid-aortic syndrome: A case report. Medicine (Baltimore). août 2016;95(34):e4622.

7. Pimiento JM, Teso D, Malkan A, Dudrick SJ, Palesty JA. Cancer of unknown primary origin: a decade of experience in a community-based hospital. Am J Surg. déc 2007;194(6):833‑7; discussion 837-838.

8. Motzer RJ, Rodriguez E, Reuter VE, Bosl GJ, Mazumdar M, Chaganti RS. Molecular and cytogenetic studies in the diagnosis of patients with poorly differentiated carcinomas of unknown primary site. J Clin Oncol. janv 1995;13(1):274‑82.

9. Rong R, Doxtader EE, Tull J, de la Roza G, Zhang S. Metastatic poorly differentiated monophasic synovial sarcoma to lung with unknown primary: a molecular genetic analysis. Int J Clin Exp Pathol. 25 nov 2009;3(2):217‑21.

10. Nakada K. The clinical features and prognosis of rhabdomyosarcoma: follow-up studies on pediatric tumors from the Japanese Pediatric Tumor Registry 1971-1980. Part II. Committee of Malignant Tumors, Japanese Society of Pediatric Surgeons. Jpn J Surg. sept 1990;20(5):503‑9.

11. Kuttesch JF, Parham DM, Kaste SC, Rao BN, Douglass EC, Pratt CB. Embryonal malignancies of unknown primary origin in children. Cancer. 1 janv 1995;75(1):115‑21.

12. Backer A, Mount SL, Zarka MA, Trask CE, Allen EF, Gerald WL, et al. Desmoplastic small round cell tumour of unknown primary origin with lymph node and lung metastases: histological, cytological, ultrastructural, cytogenetic and molecular findings. Virchows Arch. févr 1998;432(2):135‑41.

13. Morandi S, Manna A, Sabattini E, Porcellini A. Rhabdomyosarcoma presenting as acute leukemia. J Pediatr Hematol Oncol. août 1996;18(3):305‑7.

14. Fitzmaurice C, Cornett DD, Spier BJ, Pfau P. Metastatic pancreatic small-cell carcinoma presenting as acute pancreatitis. J Clin Oncol. 20 déc 2010;28(36):e748-749.

15. Yim JJ, Kang GH, Heo DS, Kim NK. A spindle cell tumor of unknown origin and diffuse bone marrow involvement in a patient with hypercalcemia. Tumori. déc 1999;85(6):526‑9.

16. Kuhel WI, Monhian N, Shanahan EM, Heier LA. Epithelioid sarcoma of the neck: a rare tumor mimicking metastatic carcinoma from an unknown primary. Otolaryngol Head Neck Surg. déc 1997;117(6):S210-213.
